# Walking Exercise Reduces Postprandial Lipemia but Does Not Influence Postprandial Hemorheological Properties and Oxidative Stress

**DOI:** 10.3390/metabo12111038

**Published:** 2022-10-28

**Authors:** Ching-Lin Wu, Tsung-Jen Yang, Min-Huan Wu, Hong-Jen Liang, Yi-Liang Chen, Shey-Lin Wu, Chih-Hui Chiu

**Affiliations:** 1 Graduate Institute of Sports and Health Management, National Chung Hsing University, Taichung 402202, Taiwan; 2 Department of Physical Education, National Taiwan Normal University, Taipei 106209, Taiwan; 3 Senior Wellness and Sport Science, Tunghai University, Taichung 404, Taiwan; 4 Department of Food Science, Yuanpei University of Medical Technology, Hsinchu City 30015, Taiwan; 5 Graduate Institute of Sports Training, University of Taipei, Taipei 11153, Taiwan; 6 Department of Neurology, Changhua Christian Hospital, Changhua 500209, Taiwan; 7 Department of Electrical Engineering, National Changhua University of Education, Changhua 500209, Taiwan; 8 Graduate Program in Department of Exercise Health Science, National Taiwan University of Sport, Taichung 404401, Taiwan

**Keywords:** high-fat meal, hemorheology, blood viscosity, endurance exercise, red blood cell aggregation

## Abstract

A higher postprandial triglycerides response and hemorheological abnormalities may increase the incidence of metabolic disorders and negatively interfere with the aging process. A single session of preprandial endurance exercise was found to be effective in reducing triglyceride levels after a high-fat diet. However, whether the exercise-induced reduction in postprandial triglyceride levels influences hemorheological indicators remains unknown. This study aims to investigate the effects of postprandial lipemia on hemorheological properties and oxidative stress. Eight healthy young male participants completed two experimental trials. On day 1, the participants were randomly assigned to walk for 1 h at 50% VO_2_max (EE trial) or rest (CON trial). On day 2, participants rested and consumed a high-fat meal in the morning. Results: The postprandial area under the curve (AUC) of plasma TG concentration was significantly lower in EE compared to CON (EE: 9.2 ± 1.9; CON: 10.9 ± 1.7 mmol/L·h^−1^; *p* = 0.013; Cohen’s *d* = 0.036). No significant difference was observed in hemorheological properties and MDA (*p* > 0.05). Endurance exercise effectively decreased postprandial TG concentration but did not influence the postprandial hemorheological properties and oxidative stress indicators.

## 1. Introduction

Hemorheological abnormalities, including an increase in plasma and blood viscosity, an increase in red blood cell (RBC) aggregation, and a reduction in RBC deformability, may increase the incidence of metabolic disorders or cardiovascular diseases [1,2], and harm the vascular aging process [3]. Transient hemorheological abnormalities are likely to influence peripheral vascular circulation and oxygen transport [4], whereas long-term hemorheological abnormalities may increase the probability of atherosclerosis and cardiovascular disease [5,6]. Therefore, improving the hemorheological abnormalities may decrease the decline of biological functions and senescence.

The blood triglyceride level has been suggested to influence hemorheological properties. Contreras et al. (2004) investigated the relationship between blood lipid levels and various hemorheological variables in 112 healthy participants and they found that blood lipid significantly influenced hemorheological properties. Specifically, the plasma triglyceride level correlated significantly and positively with blood viscosity; it was also significantly correlated with RBC deformability and was significantly and positively correlated with RBC aggregation [7]. Aloulou et al. (2006) also observed a significant and positive correlation between plasma triglyceride and blood viscosity; however, they found that plasma triglyceride was not significantly correlated with plasma viscosity, RBC deformability, and RBC aggregation [8]. Patients with high triglyceride levels are often associated with elevated low-density lipoprotein (LDL) levels, excessively high levels of free fatty acids, or an overly low level of high-density lipoprotein (HDL). The effects of these indicators on blood viscosity have yet to be determined. It is therefore necessary to explore the relationship between blood triglyceride and hemorheological properties in order to elucidate crucial influencing factors. 

The consumption of a high-fat meal is conceived as having an interfering effect on hemorheological properties, causing hemorheological disorders [9]. Previous animal studies have reported that the ingestion of a high-fat meal causes an increase in blood and plasma viscosity, an increase in RBC aggregation, and a decrease in RBC deformability [9,10]. A human study observed that ingestion of a high-fat meal prompts a rise in RBC aggregation due to an increase in blood lipid concentration [11]. Another study reported an increase in RBC aggregation 2 h after ingestion of a high-fat meal [12]. The effect of a high-fat meal on postprandial blood lipid level lasts for 6 h or longer, indicating that investigating hemorheological changes only 2 h after a meal is probably inadequate. 

The consumption of a high-fat meal induced postprandial lipemia phenomenon had been suggested to increase lipid peroxidation and may raise postprandial oxidative stress [13]. Oxidative stress was defined as the reactive oxygen and nitrogen species overwhelming cellular antioxidant defenses [14]. The high-fat meal increases lipid peroxidation, which can be measured by the percent change in malondialdehyde (MDA) [15]. The increase in the production of MDA has been linked to increased oxidative stress, and as harmful to the circulatory system, and results in various health disorders [16]. In contrast, exercise increases the reactive oxygen species and promotes oxidative stress during exercise, however, not only endurance exercise [17,18] but resistance exercise [19] improves the antioxidant defense system and leads to a decrease in postprandial oxidative stress. Exercise enhances antioxidant enzyme activity [20] and increases triglyceride clearance [18], which may be potential mechanisms for increased antioxidant capacity.

A single session of preprandial walking exercise at the intensity of 50% VO_2_max was found to be effective in reducing triglyceride levels after a high-fat diet [21,22]. However, whether an exercise-induced reduction in postprandial triglyceride levels influences hemorheological indicators remains unknown. Oxidative stress was also found as having an effect on hemorheological indicators [23]. Ingestion of a high-fat diet significantly elevates oxidative stress in the body, but the effect of a high-fat diet on postprandial hemorheological properties is also unclear. This study, therefore, aims to investigate the effects of postprandial lipemia on hemorheological properties, and to elucidate whether postprandial hemorheological properties are affected following an exercise-induced reduction in postprandial triglyceride levels.

## 2. Materials and Methods

### 2.1. Participants

Eight healthy male participants (age: 21.9 ± 0.5 years; height: 1.73 ± 0.02 m; body weight: 74.2 ± 4.4 kg) were recruited for this study. All participants exercise regularly but did not undertake any professional exercise training. Before the experiment, questionnaires were used to determine the participants’ lifestyles, health conditions, and exercise frequency. 

The inclusion criteria were: (i) healthy male adult, (ii) regular exercisers (>2 times per week), and (iii) free of high blood pressure, heart diseases, joint diseases, and other diseases under medication. The exclusion criteria were: (i) underage, (ii) not regular exercisers in the past 3 months, (iii) professional exercise/sports training experience, and (v) with diseases under medication. The participants were informed of the experimental procedures and potential risks of the experimental process and subsequently signed the Informed Consent Form after having fully understood the terms and agreed to participate in this study. This study was approved by the Institutional Review Board of the National Taiwan University of Sport (NTUPES-HSC-100-07). This study follows the principles of the Declaration of Helsinki.

### 2.2. Experimental Design

**Figure 1:**
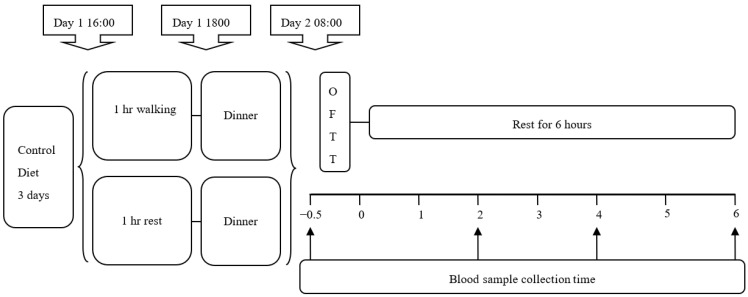
Study design. Subjects completed two experimental trials: endurance exercise trial (EE), the subjects completed the endurance exercise of 1-h 50% VO_2_max walking in the afternoon of the first day of the experiment; in the control trial, subjects were rested. On the next morning of the experiment, the subjects were given a high-fat meal and a blood sample was collected (↑). OFTT: oral fat tolerance test.

All participants completed two 2-day trials in a randomized order. The participants were randomly assigned to an endurance exercise (EE) trial or control (CON) trial. The EE trial performed a 1 h-walking exercise at 50% VO_2_max, and the CON trial was rested in the laboratory. The exercise type and intensity had been noted to decrease the postprandial TG concentration, which may be a potential mechanism that could lead to a decrease in oxidative stress. All participants exercised or rested on the afternoon of the first day, and then on the morning of the second day, the participants returned to the laboratory where they received venipuncture for fasting blood collection, after which they ingested a high-fat diet and rested while under observation for 6 h. The experimental procedure is shown in Figure 1.

### 2.3. Pretest

Prior to the experiments, all participants had to undergo a submaximal and a graded maximal exercise test to measure their VO_2_max, which were then employed to calculate the exercise intensity that represented 50% VO_2_max and the amount of energy expenditure following 1 h of exercise. Both tests were completed on the same day. A submaximal exercise test was performed first, after which the participants rested for approximately 40 min to 1 h before undergoing the VO_2_max test.

#### 2.3.1. Submaximal Exercise Test

The participants were required to walk on a treadmill at a constant speed with a gradually increasing gradient. First, the participants walked at a speed they were comfortable with, approximately 5–6 km/h, starting at 0 degree incline and increasing 2.5 degrees every 3 min for a total of four stages and 12 min. The breath-by-breath gas analysis was performed by a gas analyzer (Vmax Series 29C, SensorMedics, CA, USA). The results were used to calculate the exercise intensity and energy expenditure.

#### 2.3.2. Graded Maximal Exercise Test

The participants were required to walk on a treadmill at a constant speed with a gradually increasing gradient. Treadmill speed was set to 6–7 km/h, ranging between walking and running speed, depending on the participants’ physical capacity. At the beginning of the test, the participants started at a 0 degree incline, increasing 2.5 degrees every 3 min and walking until volitional fatigue. The maximum oxygen uptake at volitional fatigue was recorded to estimate the exercise intensity during formal testing [21,22]. 

After both the submaximal exercise test and VO_2_max tests were completed, a regression approach was used to substitute 50% VO_2_max in the four-stage gradients and oxygen levels measured from the non-VO_2_max test, then the gradient value was calculated at 50% VO_2_max. The regression of 50% VO_2_max and walking speed calculated in the submaximal exercise test had been used to calculate the exercise intensity of the participants. A similar protocol has been described in the previous study [21].

### 2.4. Experimental Process

All participants underwent two days of the main experiment at least 7 days after the pretest. Three days prior to the first experiment, the participants had to record the food they ingested. They were required to eat the same food before another trial. Three days prior to the tests, the participants had to stop exercising and avoid drinking alcoholic beverages or taking any medication, which would otherwise influence the result of this study. 

At 5–6 p.m. on the first day of the experiment, the participants arrived at the laboratory. The EE group underwent a 1 h-walking exercise at 50% VO_2_max, while the CON group rested in the laboratory. The walking speed and inclination were calculated from the pretest. At approximately 6–7 p.m. after the participants finished exercising, they consumed a standard dinner with 692 kcal; 50% energy from carbohydrates, 32% from fats, and 18% from protein. 

The participants returned to the laboratory at 8:30 a.m. the next day, and after resting for a short while, their fasting blood samples were collected via venipuncture. The participants were then given a high-fat meal and rested for 6 h after eating. Thereafter, postprandial blood samples were collected 2 h, 4 h, and 6 h after the meal to examine the changes in hemorheological properties during this period.

### 2.5. Oral Fat Tolerance Test (OFTT)

Designed by a professional nutritionist, the meal provided for the oral fat tolerance test (OFTT) was composed of toast, butter, cheese, muesli, and fresh cream. The meal provided 1.2 g of fat per kg body weight, 1.1 g of carbohydrate, 0.33 g of protein, and 16.5 kcal of energy. All meal ingredients were purchased from the same supermarket, and information on the ingredients was based on the packaging labels. The same diet has been accepted in previous studies [21].

### 2.6. Blood Collection

In this study, 10mL blood samples were collected using a cannula (cannula Venflon 20G, Helsingborg, Sweden) and three-way connector (Connecta Ltd., Helsingborg, Sweden). Samples were collected 2, 4, and 6 h after a meal. The catheter was then cleaned using 10 mL of isotonic saline to prevent blood coagulation in the tube. The blood samples were collected into a collection tube containing ethylenediaminetetraacetic acid (EDTA). Cell counter (Sysmax KX-21N, Kobe, Japan) was employed for the hematocrit test, after which 2 mL of blood was extracted for use. The remaining blood samples were centrifuged for 20 min at 500× *g* at 4 °C, and the blood plasma was extracted, retaining the blood cells at the bottom of the tube. A portion of the extracted blood plasma was stored at −80 °C, and the other portion was used for hematocrit adjustment in hemorheological examination. 

### 2.7. Blood Biochemical Analysis

The blood biochemical analysis methods had been published in the previous study [24]. A fully automated biochemical analyzer (7020, Hitachi, Tokyo, Japan) and commercial reagent (GOD-PAP method, Randox, Ireland) were adopted to analyze the levels of triglyceride, HDL-C, total cholesterol, and total protein (TP) in the blood plasma. The detailed formula is as follows:

LDL-C = TC − (TG/5 + HDL-C)

The Enzyme-linked immunoassay (ELISA) method and commercial reagent (Northwest, Vancouver, WA, USA) were used to analyze the plasma concentration of MDA [25].

### 2.8. Blood and Plasma Hemorheological Analysis

The method adopted by Tai et al. was used to perform the hemorheological analysis [10]. To advance the analysis of blood and plasma viscosity under shear rates of 75,300 and 525 mPa, the blood samples were brought to 37 °C and adjusted to contain 45% plasma and 55% red blood cells. A RheoStress 1 double-cone viscometer (HAAKE Mess-Technik, Karlsruhe, Germany) was used to conduct all analyses of blood and plasma viscosity. 

A laser diffraction device (laser-assisted optical rotational cell analyzer, USA) was used to analyze RBC deformability. The centrifuged red blood cells were added to phosphate-buffered saline (PBS) and 5.5% polyvinylpyrrolidone (PVP) solution and heated to 37 °C. Following this, 0.7 mL of the diluted solution was transferred to a transparent U-shaped plastic beam traversing plate. To display the compressed, oval-shaped RBCs on a computer monitor, the plate was then exposed to 30 Pa and laser light illumination. The computer automatically set the longer length of the oval-shaped RBC as A and the shorter length as B. The deformability index (DI) under a shear stress of 30 Pa was calculated using DI= (A − B)/(A + B). 

The laser diffraction method (laser-assisted optical rotational cell analyzer, LORCA, RR Mechatronics, Hoorn, The Netherlands) and dynamical parameters from a syllectogram were used to analyze RBC aggregation. 0.5 mL of EDTA-containing blood samples were transferred to a U-shaped plastic beam traversing plate, and following the transfer, to obtain the degree of RBC aggregation, a laser beam was applied to display the image on a computer monitor for syllectogram analysis.

### 2.9. Statistical Analysis

All data were presented as mean ± standard deviation. Two-way ANOVA with repeated measures was employed to analyze the difference in blood triglyceride levels and hemorheological properties between each group and at different time points. A significant difference with respect to group or time necessitates post hoc comparison using the Bonferroni method. Significance is achieved when *p* < 0.05. A G*power 3 [26] with an alpha value of 5% and a power of 0.8 was used to calculate the sufficient sample size of 8 participants based on the triglyceride AUC data [24].

## 3. Results

### 3.1. Treadmill Walking

The VO_2_max result from the graded maximal exercise test was 46.9 ± 8.7 mL/kg/min. The walking speed at 50% VO_2_max for 60 min was calculated by a regression equation from the result of the 16-min submaximal oxygen uptake test, which was 6.0 ± 0.1 km/h. The inclination was 8.0 ± 2.0 %. The energy expenditure for the 60 min walking exercise was 515.2 ± 45.0 kcal. 

### 3.2. Triglyceride Level

**Figure 2:**
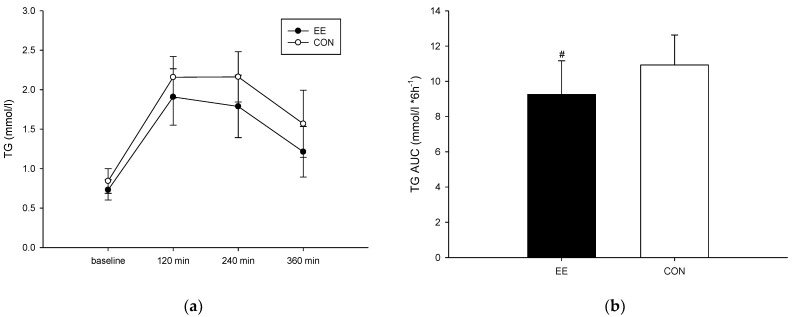
Postprandial plasma triglyceride concentrations (**a**) and the area under the curve (**b**) on the control (CON) (○) endurance exercise (EE) (●) trials. Values are mean ± SD, n = 8. # EE was significantly lower than those for the CON.

Four hours and six hours after ingestion of a high-fat diet, the triglyceride level of the EE group (Figure 2a) was not significantly different than that of the CON group (interaction, *p* = 0.431; trial, *p* = 0.014; time, *p* < 0.001). Regarding the area under the curve (AUC), the triglyceride AUC of the EE group was significantly lower than that of the CON group (*p* = 0.013) (Figure 2b). The effect size (Cohen’s dz) was 0.36 for the triglyceride AUC.

### 3.3. Blood and Plasma Viscosity

**Figure 3:**
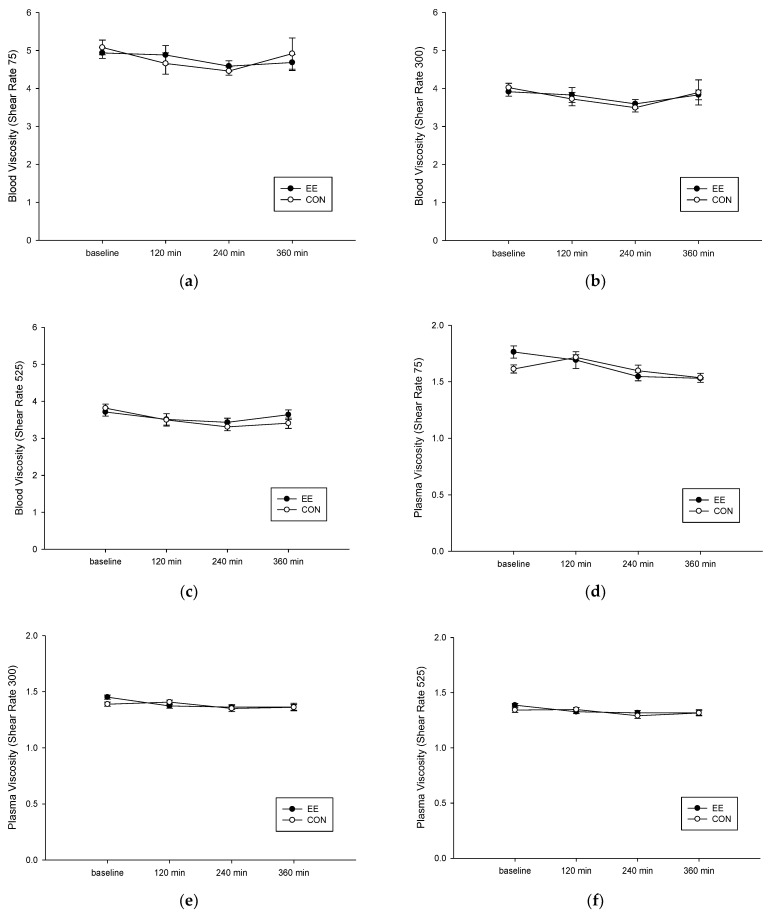
Postprandial blood and plasma viscosity under shear rate of 75 (**a**,**d**), 300 (**b**,**e**), and 525 (**c**,**f**) mPa on the control (CON) (○) endurance exercise (EE) (●) trials. Values are mean ± SD, n = 8.

Following ingestion of a high-fat diet, no difference was observed in the blood and plasma viscosity of the two groups. When the shear rate was 75 mpa.s, the blood viscosity of the EE group was significantly reduced (interaction, *p* = 0.487; trial, *p* = 0.954; time, *p* = 0.194; Figure 3a). When the shear rate was 300 mpa.s, the 4 h-postprandial blood viscosity of the CON group was significantly reduced (interaction, *p* = 0.286; trial, *p* = 0.420; time, *p* = 0.023; Figure 3b). When the shear rate was 525 mpa.s, the 4 h-postprandial blood viscosity of the CON group was significantly reduced (interaction, *p* = 0.352; trial, *p* = 0.887; time, *p* = 0.042; Figure 3c). When the shear rate was 75 mpa.s, the plasma viscosity of the EE group was significantly reduced (interaction, *p* = 0.183; trial, *p* = 0.725; time, *p* = 0.004; Figure 3d). When the shear rate was 300 mpa.s, the 4 h-postprandial plasma viscosity of the CON group was significantly reduced (interaction, *p* = 0.181; trial, *p* = 0.383; time, *p* = 0.054; Figure 3e). When the shear rate was 525 mpa.s, the 4 h-postprandial plasma viscosity of the CON group was significantly reduced (interaction, *p* = 0.184; trial, *p* = 0.384; time, *p* = 0.008; Figure 3f). 

### 3.4. The RBC Aggregation

**Figure 4:**
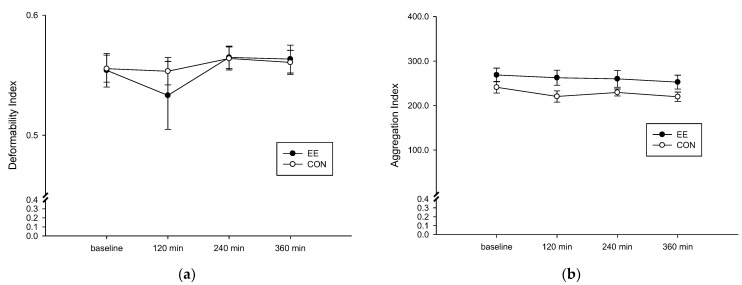
Fasting and postprandial 6 h of red blood cell deformation degree (**a**) and aggregation degree (**b**) on the control (CON) (○) and endurance exercise (EE) (●) trials. Values are mean ± SD, *n* = 8.

The RBC deformation degree of the EE group (Figure 4a) was not significantly different than that of the CON group (interaction, *p* = 0.510; trial, *p* = 0.663; time, *p* = 0.345). Following ingestion of a high-fat diet, no difference was observed in the red blood cell aggregation degree of the two groups (interaction, *p* = 0.926; trial, *p* = 0.034; time, *p* = 0.241; Figure 4b).

### 3.5. Total Cholesterol, HDL, LDL, MDA, and TP Levels in the Blood Plasma

Table 1 shows the total cholesterol, HDL, LDL, malondialdehyde (MDA), and TP levels in the blood plasma. The results indicated that both groups exhibited non-significant differences. Regarding time points, the postprandial HDL concentration was significantly reduced in both groups. The plasma MDA results revealed that the two groups did not present significant differences.

## 4. Discussion

The results of this study showed that endurance exercise could significantly lower postprandial blood lipid levels, but did not influence the postprandial hemorheological properties and oxidative stress indicators in the healthy male normal-weight participants. Postprandial triglyceride in the blood is possibly not a crucial factor influencing postprandial blood viscosity. This study also found that postprandial blood and plasma viscosity slightly decrease after a high-fat meal. The RBC aggregation of the group undertaking endurance exercise was significantly higher than that of the control group. 

Triglyceride in the blood is possibly not a crucial factor influencing postprandial blood viscosity. There were other factors that influenced blood and plasma viscosity rather than triglyceride itself. In a previous study, Babu (2009) investigated the relationship between blood triglyceride and blood viscosity in patients with high blood lipid levels and found that both factors presented a significant positive correlation [27]. However, in patients with high triglyceride levels, this is often accompanied by excessively high levels of total blood cholesterol, LDL, and fibrinogen [28]. It is therefore necessary to explore the relationship between blood triglyceride and hemorheological properties in order to elucidate crucial influencing factors. The result in this study is consistent with the findings reported by Irace et al. (2009) who observed 315 patients with high blood lipid levels as well as 95 healthy subjects and found a significant positive correlation between LDL-C in the blood and plasma viscosity, and that blood triglyceride does not influence blood viscosity [1]. In another study, Carallo et al. (2012) determined a significant relationship between blood LDL-C and blood viscosity. Although the research data did not indicate a significant correlation between LDL and blood and plasma viscosity, the data clearly showed that blood triglyceride is possibly not the main factor influencing blood and plasma viscosity [29]. In this study, one hour of walking exercise successfully reduced the postprandial triglyceride concentration but did not significantly affect postprandial hemorheological properties. Therefore, we conclude that postprandial triglyceride concentration is not the main factor affecting postprandial hemorheological properties after a high-fat meal.

Another possible reason the intervention did not affect the blood viscosity after the consumption of a high-fat meal is that endurance exercise did not affect the MDA level. Oxidative stress had been found to impair blood viscosity and rheological properties [23]. The consumption of a high-fat meal expands the magnitude of blood oxidative stress levels [13]. However, blood oxidative stress levels decrease with the 60 min endurance exercise or 10 times 60 s sprints intervention [17], or 45 min of moderate- and high-intensity endurance exercise [18]. In this study, in addition to the exercise intensity (50% VO_2_max walking exercise) being lower compared to other studies [17,18], the energy expenditure (515.2 ± 45.0 kcal) is higher in this study than the previous study (306.2 ± 28.2 kcal) [18]. The higher energy expenditure during exercise had been noted to decrease oxidative stress after a high-fat meal [30]. Therefore, we speculate that the participant population may be the reason for the lack of significance of the experimental results. The participants we included were normal-weight, healthy young adult males. The normal-weight participants had lower MDA responses compared to obese participants after the consumption of a high-fat meal [13]. The healthy fitness participants were associated with a higher antioxidant capacity [31]. The health and fitness state of the participants may be an explanation as to why the MDA, and blood viscosity did not differ among trials.

This study determined that a single high-fat diet did not influence RBC deformability, however, postprandial oxidative stress might influence RBC deformability [23]. Therefore, this study attempted to elucidate the effect of oxidative stress following a high-fat diet, and the relationship between oxidative stress and RBC deformability based on the body’s response to MDA—the product of oxidation following the attachment of free radicals to body tissues. The results revealed that there was no difference in MDA levels between the two groups, and MDA levels did not increase substantially after a meal. This study, therefore, verified that a single session of exercise and a high-fat diet did not influence RBC deformability. Future studies should investigate whether a prolonged period of high-fat intake or an extended period of exercise significantly influences RBC deformability and cholesterol level in RBC membranes.

RBC aggregation does not have any influence despite the simulation of a high-fat diet. We suggested that the lower level of postprandial triglyceride (compared with that adopted by the research team of Ehime University) might not cause a significant increase in RBC aggregation [11]. In this study, the 2 h-postprandial triglyceride level measured 168–207 mg/dL on average, which is lower than the 276–191 mg/dL reported by the research team of Ehime University. This discrepancy suggests that lower levels of plasma triglyceride might not exert any influence on RBC aggregation. Although blood fibrinogen was not detected, no studies have reported that a single session of exercise or a single high-fat meal would influence blood fibrinogen level. In this study, the total plasma protein level in both groups and at different time points did not differ significantly. The present study, therefore, verified that under low triglyceride levels, a difference in the concentration of triglyceride did not influence RBC aggregation.

In this study, it was revealed that the RBC aggregation of the group undertaking endurance exercise was significantly higher than that of the control group, and the reason behind this could not be explained. Although previous studies showed an increase in RBC aggregation following endurance exercise, the duration of the increase was generally brief, and the RBC aggregation returned to the initial level following recovery. Transient increase in RBC aggregation after exercise could be attributed to the effect of total protein concentration in the blood plasma [12]. However, no difference in total protein concentration was observed in this study. Therefore, there are no current data to explain the cause of the increase in RBC aggregation in the endurance exercise group.

The participants recruited in this study were healthy males of normal weight. The healthy adults had lower blood viscosity [1], lower RBC aggregation, and higher RBC deformability [8]. The normal-weight participants had lower MDA responses compared to obese participants after consumption of a high-fat meal [13]. It is not certain that the results of this study would apply to overweight, obese, or at-risk populations. However, the walking exercise intervention successfully decreases the postprandial triglyceride response after a high-fat meal. These results clearly suggested that endurance exercise decreases the postprandial triglyceride response but the reduction of the triglyceride response may not influence postprandial hemorheological properties and oxidative stress after a high-fat meal. Further research is required to investigate the influence of blood-related factors on hemorheological properties.

## 5. Conclusions

The results of this study revealed that endurance exercise could significantly lower postprandial blood lipid concentration and may decrease the decline of biological functions and senescence. However, the decrease in postprandial triglyceride response did not influence the postprandial hemorheological properties and oxidative stress indicators. In addition, postprandial triglyceride concentration was not the main factor influencing hemorheological properties. Subsequent studies should extensively investigate the influence of blood-related factors on hemorheological properties.

## Figures and Tables

**Table 1:** The total cholesterol (TCHO), high-density lipoprotein (HDL), low-density lipoprotein (LDL), malondialdehyde (MDA), and total protein (TP) levels in the blood plasma.

	Baseline	OFTT120	OFTT240	OFTT360	*p* Value (Interaction)
**TCHO (mmol/L)**					
EE	4.39 ± 0.15	4.13 ± 0.10	4.20 ± 0.12	4.14 ± 0.11	0.714
CON	4.44 ± 0.12	4.28 ± 0.16	4.34 ± 0.11	4.39 ± 0.11	
**HDL (mmol/L)**					
EE	2.41 ± 0.16	2.28 ± 0.16	2.21 ± 0.15	2.34 ± 0.17	0.032
CON	2.55 ± 0.17	2.34 ± 0.17	2.24 ± 0.14	2.31 ± 0.15	
**LDL (mmol/L)**					
EE	0.73 ± 0.13	1.91 ± 0.36	1.79 ± 0.40	1.21 ± 0.32	0.431
CON	0.84 ± 0.16	2.16 ± 0.26	2.16 ± 0.32	1.57 ± 0.42	
**TP (mmol/L)**					
EE	7.1 ± 0.2	7.0 ± 0.2	7.1 ± 0.2	6.9 ± 0.2	0.304
CON	7.2 ± 0.2	6.8 ± 0.2	7.0 ± 0.3	7.1 ± 0.2	
**MDA (μM)**					
EE	4.02 ± 1.9	5.63 ± 2.6	5.48 ± 2.5	4.22 ± 2.4	0.526
CON	4.66 ± 2.0	5.25 ± 1.8	5.02 ± 2.1	5.26 ± 2.6	

## Data Availability

All relevant materials are presented in the present manuscript.
